# The effect of technical details of percutaneous catheter drainage on the clinical outcomes of infected necrotizing pancreatitis patients

**DOI:** 10.3906/sag-1805-111

**Published:** 2019-08-08

**Authors:** Zhi-Hua ZHANG, Yi-Xuan DING, Yu-Duo WU, Chong-Chong GAO, Fei LI

**Affiliations:** 1 Department of General Surgery, Beijing Xuanwu Hospital, Capital Medical University, Beijing P.R. China; 2 Department of Hepatobiliary Surgery, Beijing Chaoyang Hospital, Capital Medical University, Beijing P.R. China

**Keywords:** Infected necrotizing pancreatitis, percutaneous catheter drainage, videoscopic assisted retroperitoneal debridement, prognosis

## Abstract

**Background/aim:**

This study aimed to investigate the effect of technical details of percutaneous catheter drainage (PCD) on the clinical outcomes of patients with infected necrotizing pancreatitis (INP).

**Materials and methods:**

A total of 44 INP patients treated in our hospital from October 2013 to October 2015 were included. The correlations of the first PCD treatment data and the clinical outcomes were analyzed.

**Results:**

The number of catheters was positively correlated with hospital readmission (r = 0.335, P = 0.032). Receiver operating characteristic curve analysis showed that patients with ≥ 3 catheters were more likely to have hospital readmission. Patients with pleural effusion undergoing thoracentesis were more likely to have new intensive care unit admission (P = 0.025) and bleeding in need of intervention (P = 0.032). Patients with more effusion regions had higher incidences of mortality (P = 0.012) and new intensive care unit admissions (2.44 ± 1.03 vs. 1.88 ± 0.80; P = 0.059). Patients with PCD only were less likely to have new intensive care unit admissions (22.22% vs. 54.55%; P = 0.038) than those with PCD + small incision or/and videoscopic assisted retroperitoneal debridement.

**Conclusion:**

Number of catheters greater than three was associated with unfavorable outcomes of PCD treatment in INP patients. Patients that received PCD treatment only had better outcomes.

## 1. Introduction

Acute necrotizing pancreatitis is the most severe form of acute pancreatitis characterized by focal macroscopic or diffuse necrosis in pancreatic parenchyma or/and the peripancreatic tissues [1]. Approximately up to 30% of necrotizing pancreatitis patients develop a secondary infection in pancreatic necrosis [2], which is known as infected necrotizing pancreatitis (INP) [3]. 

With the advancement of minimally invasive techniques, the “step-up” approach has been developed for INP treatment [4]. The step-up approach adopts minimally invasive interventions to control the source of pancreatic infection, and a progressive strategy to remove the necrotic and infected tissues [4]. Percutaneous catheter drainage (PCD) is the first step in the step-up approach. If the clinical symptoms are not relieved or poor drainage is present at 72 h after PCD treatment, other drainage locations or multiple drainages can be considered for the PCD treatment. A metaanalysis including 384 patients from 11 studies shows that 55.7% of patients with necrotizing pancreatitis can be treated with PCD without the need for surgical necrosectomy [5]. 

Even though the step-up approach is widely accepted as the primary treatment strategy for INP, there is no consensus on the technical details of PCD such as the timing of primary PCD, number of catheters, drain size, and total number of drainage procedures [6]. On the other hand, the reported therapeutic outcomes after primary PCD for INP also markedly vary among studies [7–11], which might be attributed to the different PCD strategies utilized. The impact of different PCD strategies on the clinical outcomes remains not fully understood. Therefore, the purpose of this study was to investigate the effect of technical details of PCD on the clinical outcomes of INP patients.

## 2. Materials and methods

### 2.1. Patients

A total of 44 INP patients treated in our hospital from October 2013 to October 2015 were included in this study. Classification, staging, pathological type, severity, and local complications of acute pancreatitis were defined and diagnosed according to the 2012 revision of the Atlanta Classification of acute pancreatitis [12]. Inclusion criteria were: 1) suspected or confirmed diagnosis of INF; 2) PCD as initial treatment; 3) combined with pancreatic effusion of >100 mL. Exclusion criteria included: 1) mild acute pancreatitis; 2) PCD was not the initial treatment; 3) pancreatic pseudocyst. 

### 2.2. Data collection

The following baseline characteristics were collected: sex, age, and disease etiology. Also, clinical parameters including length of hospital stay, length of intensive care unit (ICU) stay, and total number of hospitalizations were collected. 

The following information about the first PCD treatment was collected: timing of first PCD procedure (between necrotizing pancreatitis onset and PCD treatment), number of catheters, drain locations, total number of drainage procedures, maximal catheter size, total number of effusion regions (including pleural effusion, splenic effusion, hepatic effusion, iliac fossa effusion, peripancreatic effusion), receiving abdominocentesis or thoracentesis. 

The clinical outcomes after PCD collected for evaluation were open necrosectomy, new ICU admission, hospital readmission, bleeding in need for intervention, new-onset (multi)organ failure, and mortality.

### 2.3. Statistical analysis

Continuous data were presented as the mean ± standard deviation (SD). Means were compared by Student’s independent t-test. The Mann–Whitney tests were used if normality of continuous data was not assumed. Categorical data were presented as number and percentage and were compared by chi-square test or Fisher’s exact test if the expected value was found lower than 5. Correlation coefficient analyses were adopted to observe the associations between independent variables. ROC analysis was used to analyze the association between independent continuous variables and major outcomes. All statistical analyses were performed using IBM SPSS Version 20 (SPSS Statistics V20, IBM Corporation, Somers, New York, USA). Two-tailed significance level was set at P < 0.05. 

## 3. Results

### 3.1. Patients’ demographic and clinical characteristics

A total of 44 patients receiving PCD (mean age 47.39 ± 14.69 years) were included in this study, including 23 males (52.27%) and 22 females (47.73%). The demographic and clinical characteristics, as well as the outcomes, were summarized in Table 1. The mean total number of effusion regions was 2.11 ± 0.97. The mean interval between NP onset and PCD was 31.67 ± 20.85 days. As shown in Table 2, all patients were treated with four different interventions, including PCD alone (45.45%), PCD + videoscopic assisted debridement (VARD) (11.36%), PCD + small incision (VARD via middle abdominal incision, 36.36%), and PCD + small incision + VARD (6.82%). As for PCD, the majority of patients (86.36%) underwent multiple catheter insertions, and the mean number of catheters was 3.30 ± 1.96. The total number of drainage procedures was 3.68 ± 2.47. There were 14 (31.82%) and 11 (25%) cases requiring abdominocentesis and thoracentesis, respectively. The mean duration of hospitalization and ICU stay were 55.80 ± 37.32 days and 18.95 ± 22.20 days, respectively.

**Table 1:** Patients’ demographic and clinical characteristics.

Parameters	Mean ±SD or N (%)
Sex	
Male	23 (52.27)
Female	21 (47.73)
Age, year	47.39 ± 14.69
Cause of pancreatitis	
Trauma	1 (2.27)
Biliary	13 (29.55)
Hyperlipidemia	7 (15.91)
Alcoholic	6 (13.64)
Post-ERCP	2 (4.55)
Others	15 (34.09)
Interval between NP onset and PCD, days	31.67 ± 20.85
1 month as cut-off	
≤1 month	28 (65.12)
>1 month	15 (34.88)
21 days as cut-off	
≤21 days	17 (39.53)
>21 days	26 (60.47)
Number of catheters, N	3.30 ± 1.96
Single or multiple	
Single catheter	6 (13.64)
Multiple catheters	38 (86.36)
3 as cut-off	
Catheters <3	27 (61.36)
Catheters ≥3	17 (38.64)
Catheter locations	
Left retroperitoneal	14 (51.85)
Right and middle retroperitoneal	13 (48.15)
Total number of drainage procedures, N	3.68 ± 2.47
Maximal catheter size, French	29.64 ± 7.60
Total number of effusion regions, N	2.11 ± 0.97
Abdominocentesis	14 (31.82)
Thoracentesis	11 (25.00)
Length of hospital stay, days	55.80 ± 37.32
Length of ICU stay, days	18.95 ± 22.20
Total number of hospitalizations, times	3.16 ± 2.06

Abbreviations: SD, standard deviation; ERCP, endoscopic retrograde cholangiopancreatography; NP, necrotizing pancreatitis; ICU, intensive care unit.

**Table 2:** Intervention methods

Interventions	N (%)
PCD	20 (45.45)
PCD + small incision	16 (36.36)
PCD + VARD	5 (11.36)
PCD + small incision + VARD	3 (6.82)
Intervention groups	
PCD only	20 (45.45)
PCD + small incision or/and VARD	24 (54.55)

PCD, percutaneous catheter drainage; small incision, videoscopic assisted debridement via middle abdominal incision; VARD, videoscopic assisted retroperitoneal debridement.

### 3.2. Clinical outcomes after PCD

The clinical outcomes after PCD were summarized in Table 3. After primary PCD treatment, 28 (68.29%) and 16 (40%) cases needed hospital readmission and new ICU admission, respectively. The mean number of hospitalizations was 3.16 ± 2.06 times. Seventeen (43.59%) cases needed open necrosectomy and 6 (14.29%) cases had bleeding needing intervention. Three (7.14%) patients had new-onset (multi)organ failure, and 4 (9.09%) patients died.

**Table 3:** Clinical outcomes after PCD

Clinical outcomes	N (%)
Need for necrosectomy	17 (43.59)
Need for new ICU admission	16 (40.00)
Need for hospital readmission	28 (68.29)
Bleeding in need of intervention	6 (14.29)
New-onset (multi)organ failure	3 (7.14)
Mortality	4 (9.09)

Abbreviations: PCD, percutaneous catheter drainage; ICU, intensive care unit.

### 3.3. Correlations between drainage characteristics and clinical outcomes

The effects of drainage characteristics on the clinical outcomes were investigated. The number of catheters was positively correlated with hospital readmission (r = 0.335, P = 0.032). Receiver operating characteristic (ROC) curve analysis showed that patients with ≥3 catheters were more likely to have hospital readmission (area under curve (AUC) = 0.705, sensitivity = 0.750, specificity = 0.538, Youden’s index = 0.288) than those with <3 catheters (P = 0.037, Figure). Patients with multiple catheters had a slight but not significant lower mortality rate as compared with those with a single catheter (7.89% vs. 16.67%; P = 0.487). Patients with necrosectomy had a smaller maximal catheter size (27.06 ± 9.11 F vs. 31.82 ± 5.72 F; P = 0.053, marginal significance). Patients with right or middle retroperitoneal drainage had a higher rate of open necrosectomy than those with left retroperitoneal drain (54.55% vs. 16.67%; P = 0.089, marginal significance).

**Figure:**
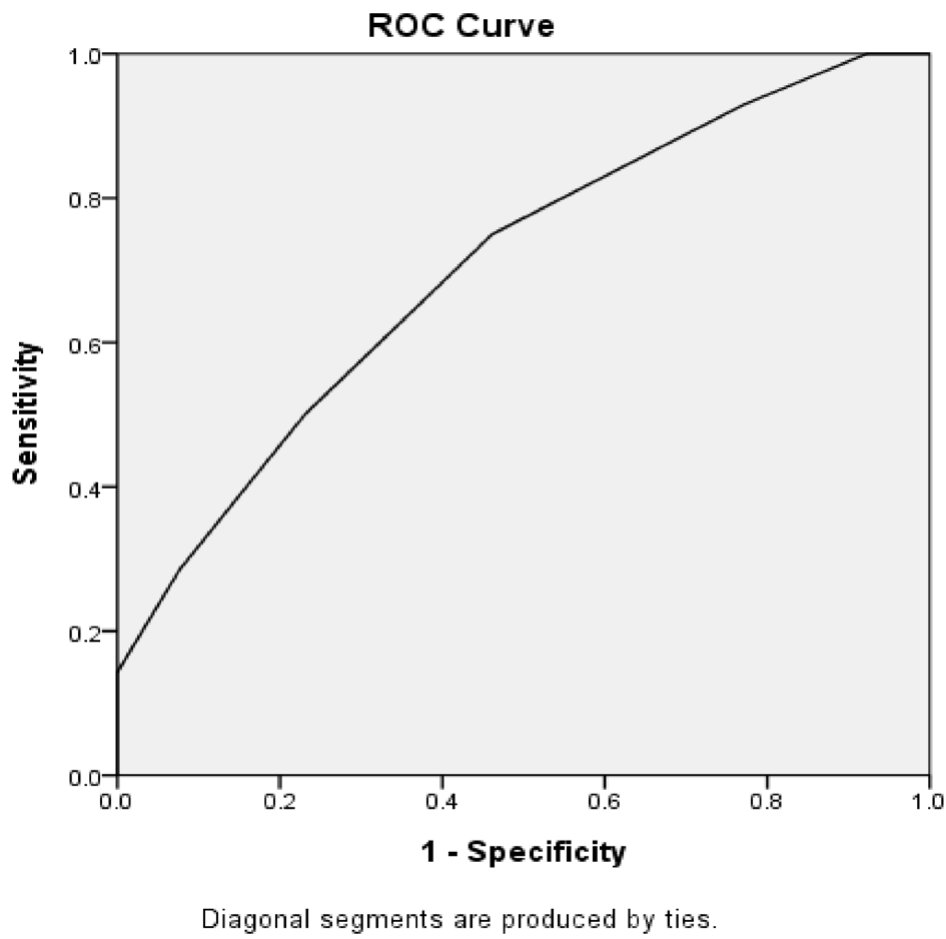
Receiver operating characteristic (ROC) curve analysis for the effect of number of catheters on hospital readmission.

### 3.4. Correlation between abdominocentesis, thoracentesis, effusion, and clinical outcomes

Patients with or without abdominocentesis had comparable clinical outcomes (all P > 0.05). However, patients with thoracentesis were more likely to have new ICU admissions (70% vs. 30%; P = 0.025) and bleeding needing intervention (36.36% vs. 6.45%; P = 0.032). Patients with more effusion regions had higher mortality (3.25 ± 0.50 vs. 2.00 ± 0.93; P = 0.012) and need for new ICU admission (2.44 ± 1.03 vs. 1.88 ± 0.80; P = 0.059).

### 3.5. Correlation between intervention methods and clinical outcomes

Next, we analyzed the association between intervention methods and clinical outcomes. The results showed that patients with PCD only had a significantly lower rate of new ICU admissions (22.22% vs. 54.55%, P = 0.038) than those with PCD + small incision or/and VARD.

The length of hospital stay was positively correlated with length of ICU stay (r = 0.476, P = 0.001) and patient’s age (r = 0.310, P = 0.040).

## 4. Discussion

Several recent studies investigating the predictive factors of the clinical outcomes of PCD have been reported. Hollemans et al. have demonstrated that male sex, multiple organ failure, increasing percentage of pancreatic necrosis, and heterogeneity of the collection are negative predictors for success of catheter drainage in infected necrotizing pancreatitis [13]. Cao et al. have demonstrated that a reduction in fluid collection by <50% following PCD, peripancreatic necrosis of >50%, and multiple organ failure are predictive factors for necrosectomy in INP patients following PCD failure [14]. Ji et al. have revealed that mean computed tomographic density of necrotic fluid collection and multiple-organ failure are independent pre-PCD and post-PCD risk factors for the need of necrosectomy following PCD. However, studies on the effect of technical details of PCD on the clinical outcomes of INP patients are limited.

 In this study, we investigated the effect of technical details of PCD on the clinical outcomes of INP patients. The results showed that there was a positive correlation between the number of catheters and hospital readmission. ROC curve analysis showed that patients with ≥3 catheters were more likely to have hospital readmission as compared with those with <3 catheters. Patients with open necrosectomy had a smaller mean maximal catheter size in primary PCD (P = 0.053, marginal significance). Patients undergoing left retroperitoneal drain were less likely to need open necrosectomy than those with right or middle retroperitoneal drain (P = 0.089, marginal significance). Patients needing thoracentesis were more likely to have new ICU admission and bleeding in need of intervention. Patients with more effusion regions had higher incidences of mortality and new ICU admissions (2.44 ± 1.03 vs. 1.88 ± 0.80; P = 0.059). Patients receiving PCD only had significantly lower rates of need for necrosectomy and new ICU admission than those with PCD + small incision or/and VARD. Taken together, our results showed that number of catheters, maximal catheter size, and thoracentesis may affect the clinical outcomes of PCD treatment in INP patients. Patients treated with PCD only had better clinical outcomes.

PCD is performed under the guide of CT, placing the catheter percutaneously to drain the necrotic tissue around the pancreas. PCD can control sepsis and effectively improve the clinical outcome of patients. Accumulating evidence has demonstrated that a considerable proportion of NP patients receiving PCD alone do not need additional necrosectomy. A randomized controlled trial by Van Santvoort et al. has reported that 15 out of 43 (35%) patients were successfully treated with PCD alone without additional necrosectomy [15]. Van Baal et al. have demonstrated a success rate of 56% (214 out of 384 patients) for PCD treatment alone [5]. In this study, 20 out of 44 INP patients (45.45%) survived with PCD therapy only, which is consistent with the notion that PCD should be used as the first step in the step-up approach and can effectively delay or even avoid necrosectomy. Our results showed that patients treated with PCD only had better outcomes, namely, lower rates of need for open necrosectomy and new ICU admission than those with PCD + small incision or/and VARD.

There is no consensus on the timing of PCD in the step-up approach. A significant variation in the timing of PCD could be observed among the reports by different centers [16], ranging from 9 to 55 days after the onset of symptoms [5]. Sugimoto et al. have shown that PCD can achieve better outcomes if proactively performed in the early stages of necrotizing pancreatitis before the development of severe sepsis [17]. In this study, the mean interval between NP onset and PCD was 31.67 ± 20.85 days. Our results showed that no correlation was found between the timing of catheter drainage and clinical outcomes. However, due to the small sample size of the current study, this finding should be validated in a study with a large sample size. The effect of the number of catheters on the clinical outcomes is rarely reported. In this study, we found a positive correlation between the number of catheters and the need for hospital readmission. Multiple catheters may slightly reduce mortality rate but increase the need for hospital readmission. This phenomenon should be attributed to the fact that patients with multiple catheters were more likely to have hospital readmission for manipulation, changing, or removal of the catheters. 

Regarding the catheter size, Bruennler et al. showed that the median drainage size and the largest drainage size have no influence on mortality [8], which is consistent with our findings. However, we found that a smaller maximal catheter size in primary PCD may lead to the need for subsequent open necrosectomy. In this study, the drainage diameter ranged from 10 French catheters to 30 French catheters. Likewise, Dougaz et al. have reported that PCD with catheter size equal to or smaller than 10 French is an independent factor associated with PCD failure [18]. A small catheter is easily obstructed, which requires manipulation or changing of the catheter and increases the need for open necrosectomy. As for drainage location, left retroperitoneal route is regarded as optimal access to the pancreatic necrosis [19], which facilitates the laparoscopic retroperitoneal necrosectomy in the following step. In this study, the preferred approach was left retroperitoneal route. Our analysis showed that patients undergoing left retroperitoneal drainage were less likely to need open necrosectomy as compared with those with right or middle retroperitoneal drainage. There are more organs on the right side than on the left side of abdominal cavity, which makes the right retroperitoneal drainage more likely to fail, leading to an increase in the need for open necrosectomy. In this study, we also found that INP patients with pleural effusion undergoing thoracentesis were more likely to have new ICU admissions and bleeding needing intervention, which may be attributed to the fact that INP patients with pleural effusion were in a state of a more severe disease. It has been shown that pleural effusion is observed in 4%–20% of patients with acute pancreatitis and is strongly associated with severity of acute pancreatitis [20]. 

It should be pointed out that this study has several limitations. Firstly, this study is a retrospective one. In addition, the sample size is relatively small. In the future, a well-designed prospective study with a large sample size should be conducted to validate the findings of the current study. 

In summary, this study showed that number of catheters greater than three was associated with unfavorable outcomes of PCD treatment in INP patients. Patients that received PCD treatment only had better clinical outcomes. Our findings should aid the understanding of the effect of technical details of PCD on the clinical outcomes of INP patients. 

## Acknowledgment

This study was funded by the Beijing Municipal Administration of Hospitals, Clinical Medicine Development project under grant [XMLX 201404].
